# Deaths in Immigration and Customs Enforcement (ICE) detention: FY2018–2020

**DOI:** 10.3934/publichealth.2021006

**Published:** 2021-01-11

**Authors:** Sophie Terp, Sameer Ahmed, Elizabeth Burner, Madeline Ross, Molly Grassini, Briah Fischer, Parveen Parmar

**Affiliations:** Keck School of Medicine, University of Southern California, Los Angeles, CA, USA

**Keywords:** immigration health, COVID-19, infectious diseases, custodial health, public health

## Abstract

**Background:**

Many civil liberties organizations have raised concerns that substandard medical care in United States Immigration and Customs Enforcement (ICE) detention facilities have led to preventable deaths. The 2018 Department of Homeland Security Appropriations Bill required ICE to make public all reports regarding in-custody deaths within 90 days beginning in Fiscal Year (FY) 2018. Accordingly, ICE has released death reports following each in-custody death since April of 2018. This study describes characteristics of deaths among individuals in ICE detention following the FY2018 mandate.

**Methods:**

Data was extracted from death reports published by ICE following the FY2018 mandate. Causes of death were categorized as suicide or medical, and medical deaths as COVID-19-related or not. Characteristics were compared between medical and suicide deaths, and among medical deaths between COVID-19-related and non-COVID-19-related deaths. Additionally, death rates per person-year and per 100,000 admissions were calculated for FY2018, 2019, and 2020 using methods from prior work evaluating deaths among detained immigrants in the United States.

**Results:**

Since April 2018, 35 individuals have died in ICE detention. The death rate per 100,000 admissions in ICE detention was 2.303 in FY2018, 1.499 in FY2019, and 10.833 in FY2020. Suicide by hanging was identified as the cause of death in 9 (25.7%), and medical causes in the remaining 26 (74.3%). Among 26 deaths attributable to medical causes, 8 (30.8%) were attributed to COVID-19, representing 72.7% of 11 deaths occurring since April 2020.

**Conclusions:**

The death rate among individuals in ICE detention is increasing amidst the COVID-19 pandemic. Potentially preventable causes of death including COVID-19 and suicide contribute to at least half of recent deaths. Findings suggest that individuals detained by ICE may benefit from improved psychiatric care and prevention measures to combat suicide, as well as increased infection control efforts to reduce mortality associated with COVID-19.

## 1. Introduction

The American Civil Liberties Union, Detention Watch Network, the National Immigrant Justice Center, and leadership from the American Medical Association have raised concerns that substandard medical care in U.S. Immigration and Customs Enforcement (ICE) detention has contributed to preventable deaths [1],[2]. In fact, a recent investigation by the Office of the Inspector General identified significant health and safety risks, along with inadequate medical care in ICE detention facilities [3]. While concerns have been raised about deaths from suicide [4] and COVID-19 [5],[6] in ICE detention, no systematic evaluation of circumstances surrounding deaths of individuals detained by ICE has been published in the peer reviewed medical literature since 2015 [7]. The 2018 Department of Homeland Security Appropriations Bill required ICE to make public all reports regarding an in-custody death within 30 days, with subsequent reporting to be completed and released within 60 days of the initial report, beginning in Fiscal Year (FY) 2018 [8]. ICE has released death reports following each in-custody death since April 2018 [9]. The goal of this study is to describe causes of and circumstances surrounding deaths among individuals in ICE detention using death reports released following implementation of the FY2018 mandate.

## 2. Methods

This exploratory case series analysis of government reports describes causes of and circumstances surrounding deaths among individuals in ICE detention using data extracted from death reports released following the FY2018 mandate. ICE death reports present demographic information including country of citizenship, immigration history, criminal history, medical history, along with circumstances surrounding death as well as the final cause of death. Deaths of individuals in ICE custody are included regardless of the location where the death occurred (federal detention facility, private detention facility, hospital, etc). To ensure completion of the dataset, the list of death reports was cross-referenced with news releases issued by ICE and immigrant advocacy organizations [10],[11]. Data was extracted from ICE-issued news releases for two deaths occurring in the final month of FY2020 for which formal death reports are pending [11]. Cause of death was categorized as suicide if found hanging immediately preceding death, or if the cause of death was listed specifically as hanging or suicide. Deaths not attributable to suicide were categorized as medical. Medical deaths were reviewed for instances where COVID-19 was specifically listed as a cause of or contributory to death. Duration of time in ICE custody prior to transfer to hospital, and whether a detained person was pronounced dead (1) by emergency medical services (EMS) in the detention center; (2) within 60 minutes of emergency department arrival; or (3) during hospital admission were recorded. These metrics were collected in order to provide context regarding acuity of illness at the time that hospital-level care was sought. Specifically, these metrics were intended to help elucidate whether detained individuals were transported to hospitals on the brink of death, thus expiring shortly after arrival or whether deaths occurred after prolonged illness or hospitalizations. For those hospitalized at the time of death, the duration of terminal hospitalization was recorded. For those receiving care at multiple hospitals (e.g. transferred between hospitals for specialized care), the total number of hospital days from terminal hospital transfer from a detention facility was recorded. Each ICE death report was systematically reviewed by two authors and data was systematically extracted.

This was an exploratory descriptive study based upon a limited number of observations not definitively powered to detect differences between groups. However, as part of the exploration, characteristics were compared between medical and psychiatric deaths, and among medical deaths caused by COVID-19 and medical deaths resulting from other causes. We used Student's t-tests to compare ages, negative binomial regressions to compare length of time in ICE custody and among those hospitalized at death, duration of hospitalization, and Fischer's exact tests for proportions. Two illustrative case studies with summaries of events as reported in death reports have been included in the supplementary exhibits to provide contextualized examples of circumstances surrounding recent deaths in ICE custody.

Additionally, death rates per person-year and per 100,000 admissions were calculated for FY2018, FY2019, and FY2020 using methods from prior work evaluating deaths among detained immigrants in the United States [7]. The number of deaths and average daily population were obtained from reports posted on the ICE website [9],[12],[13]. Statistical analyses were performed using Stata/MP13 (StataCorp. 2013. College Station, TX). Given that we analyzed publicly available documents pertaining to deceased subjects, the study was determined not to require approval by the IRB at the University of Southern California, Los Angeles.

## 3. Results

Of 38 deaths reported in ICE custody since the beginning of FY2018, 35 (92.1%) occurred after ICE began posting death reports to the ICE website in April 2018. Overall, there were 9 deaths reported in FY2018 including 6 after ICE began posting reports, 8 in FY2019, and 21 thus far in FY2020. Death rates per person-year and per 100,000 admissions are included in Table 1. The death rate per 100,000 admissions in ICE detention was 2.303 in FY2018, 1.499 in FY2019, and 10.833 in FY2020.

The mean age at death was 47.3 years (range 21.3–75.0); 32 (91.4%) were male and 3 (8.6%) were female (including one transgender woman). The deceased had been in ICE custody for a median of 36.0 days (range 1–522) at the time of death or terminal hospital transfer. Among deaths, 23 (65.7%) occurred in the hospital inpatient setting, 5 (14.3%) within an hour of emergency department arrival, and 7 (20%) in the pre-hospital setting. Among those hospitalized at the time of death, the median duration of hospitalization was 8.0 days (range 1–140). Of 35 deaths occurring after ICE began posting death reports, 26 (74.3%) causes of death were categorized as medical, and 9 (25.7%) as suicide. Characteristics of individuals that died in ICE detention during the study period are included in Table 2.

**Table 1.** Death Rates in U.S. Immigration and Customs Enforcement (ICE) Custody Fiscal Years 2018 to 2020 (n = 38).

Deaths	Year	Average Daily Population (ADP), n	Average Length of Stay (ALOS), d	Admissions per Year, n	Death Rate per Person Year	Death Rate per Person Year
9	2018	42188	39.4	390828	0.021	2.303
8	2019	50165	34.3	533826	0.016	1.499
21	2020	33724	63.5	193847	0.062	10.833

Note: This table uses methodology used by prior investigators evaluating death rate per 100,000 admissions to ICE detention [7]. Average Daily Population (ADP) and Average Length of Stay (ALOS) were obtained from reports obtained from the ICE website [12],[13]. Admissions per Year = ADP*365/ALOS. Death Rate per Person Year = (Deaths/ADP) × 100. Death Rate per 100,000 Admissions = (Deaths × 100,000)/Admissions per Year. This table includes three deaths occurring in FY2018 before ICE began routinely posting death reports to their website.

**Table 2.** Characteristics of individuals that died in ICE detention by category of cause of death.

Characteristic	By Category of Cause of Death			
	All Deaths	Suicide	Medical	P value*****
Subjects, No. (%)	35	9 (25.7)	26 (74.3)	
Age, Mean (SD) [range], y	47.3 (13.6) [21.3–75.0]	42.0 (15.9) [21.3–75.0]	49.2 (12.5) [22.6–73.0]	0.175
Gender				
Male	32 (91.4)	9 (100)	23 (88.5)	0.553
Female*	3 (8.6)	0 (0)	3 (11.5)	
ICE detention, median(IQR)[range], d**	36.0 (10–86) [1–522]	76.0 (10–119) [1–343]	32.5 (14–85) [1–522]	0.560
Location of Death, No. (%)				
Prehospital setting	7 (20.0)	6 (66.7)	1 (3.8)	<0.000
Emergency department***	5 (14.3)	1 (11.1)	4 (15.4)	1.000
Hospital inpatient setting	23 (65.7)	2 (22.2)	21 (80.8)	0.003
Hospital days, median(IQR) [range]****	8.0 (4.0–27.0) [1–140]	2.5 (1.0–4.0) [1–4]	11.0 (5.0–27.0) [1–140]	0.015

Note: *Includes one individual whose sex was listed in the death report as male but who identified as female; **Duration in ICE custody refers to the number of days in ICE custody prior to death or terminal hospital transfer; ***Deaths pronounced within one hour of arrival to hospital emergency department; ****Includes number of calendar days an individual was hospitalized following their terminal hospital transfer from detention facility, conditional on hospitalization; *****Student's t-tests were used to compare ages, negative binomial regression to compare length of time in ICE custody and among those hospitalized at death, duration of hospitalization, and Fischer's exact tests for proportions.

Among those who died of suicide, all were male, the mean age at death was 42.0 years (range 21.3–75.0), and all died by hanging. Three (33.3%) had been on suicide watch at some point during their detention. Eight (88.9%) of 9 received cardiopulmonary resuscitation (CPR) by detention staff prior to EMS arrival. In the case where CPR was not performed, detention staff found the deceased in administrative segregation hanging and unconscious 10 minutes after the last reported segregation rounds. EMS arrived and cut the individual down 15 minutes after staff discovered the individual, and reportedly did not initiate CPR. EMS was called for all cases, and pronounced death at the detention center for 6 (66.7%). Of three transported to the hospital, death was declared within an hour of arrival for one, and two were admitted with anoxic brain injury; one died within eight hours of hospital admission, and another on hospital day four after care was withdrawn. Compared to medical deaths, deaths attributable to suicide were more often declared in the pre-hospital setting (66.7% vs. 3.8%; p < 0.001). Among those hospitalized at the time of death, median hospitalization durations were shorter for those that died of suicide compared with medical causes (2.5 vs. 11.0 days; p = 0.015).

Characteristics of COVID-19-related and non-COVID-19-related medical deaths are included in Table 3. Of 26 medical deaths, 8 (30.8%) were attributed to complications of COVID-19. COVID-19 was responsible for 8 (74.2%) of 11 deaths occurring since April 2020, including all 4 deaths reported since August 2020. Among those who died of complications related to COVID-19, all were male, and the mean age at death was 56.9 years (compared with 45.7 years for those that died of other medical causes) (p = 0.032). Individuals that died from COVID-19 were all hospitalized at the time of death with a median hospitalization of 29.5 days (IQR 12.5–35.5). Among those who died of complications associated with COVID-19, four (50%) had diabetes, one (12.5%) had lymphoma, and two (25%) were elderly (including one with diabetes and chronic kidney disease). Two COVID-19-related deaths occurred in men (50 and 56 years of age) without reported history of medical conditions.

**Table 3.** Characteristics of individuals that died in ICE detention by medical sub-category of cause of death.

Characteristic	COVID-19-related	Non-COVID-19	P value*****
Subjects, No. (%)	8 (30.8)	18 (69.2)	
Age, Mean (SD) [range], y	56.9 (12.0) [34.9–73.0]	45.7 (11.4) [22.6–63.5]	0.032
Gender			
Male	8 (100)	15 (83.3)	0.529
Female*	0 (0)	3 (16.7)	
ICE detention, median (IQR) [range], d**	32.0 (17.5–94.5) [14–115]	32.5 (8–78) [1–522]	0.449
Location of Death, No. (%)			
Prehospital setting	0.0 (0.0)	1 (5.6)	1.000
Emergency department***	0.0 (0.0)	4 (22.2)	0.277
Hospital inpatient setting	8 (100.0)	13 (72.2)	0.281
Hospital days, median (IQR) [range]****	29.5 (12.5–35.5) [11–39]	6.0 (3.0–8.0) [1–140]	0.404

Note: *Includes one individual whose sex was listed in the death report as male but who identified as female; **Duration in ICE custody refers to the number of days in ICE custody prior to death or terminal hospital transfer; ***Deaths pronounced within one hour of arrival to hospital emergency department; ****Includes number of calendar days an individual was hospitalized following their terminal hospital transfer from detention facility, conditional on hospitalization; *****Student's t-tests were used to compare ages, negative binomial regression to compare length of time in ICE custody and among those hospitalized at death, duration of hospitalization, and Fischer's exact tests for proportions.

Among individuals who died of medical causes other than COVID-19, one death was pronounced at the detention facility. The details of this case are included in Supplementary Exhibit 1. Four (22.2%) were transported by EMS to hospitals where death was pronounced within one hour of emergency department arrival. These included three instances of detained men who were discovered in cardiopulmonary arrest early in the morning and transported by EMS to emergency departments where death was declared shortly after arrival. A fourth case involving a man with recent diagnosis of small bowel obstruction who went into cardiac arrest shortly after emergency department arrival is described in Supplementary Exhibit 2. An additional 13 (72.2%) died while hospitalized for a variety of cardiovascular, neurological, gastrointestinal, renal and oncological conditions.

## 4. Discussion

The death rate among individuals in ICE detention has increased seven-fold between FY2019 and FY2020 amidst the COVID-19 pandemic, while the average daily population in custody decreased by nearly a third. The death rate per 100,000 admissions has reached a record level not reported since ICE implemented Performance Based National Detention Standards in 2008 [7].

Among individuals in ICE detention, potentially preventable causes of death—including COVID-19 infection, influenza and suicide—are responsible for at least half of recent deaths.

Among individuals that died in ICE detention, the average age at death was 47.3 years which is markedly lower than reported life-expectancy for foreign-born individuals in the United States (83.9 years for men and 86.7 for women) [14]. This is concerning, particularly considering that foreign-born individuals in the United States have a 2.4-year advantage in life-expectancy relative to United States-born population [14]. Most were detained for more than a month prior to death or terminal hospital transfer, suggesting that events immediately preceding detention, such as border crossings, are unlikely to have directly contributed to premature death. Overall, 91.4% of individuals who died in ICE detention during the study period were male which likely reflects the demographics of the population detained by ICE, previously estimated to be between 85–91% male [15],[16]. Detailed demographic information on currently detained individuals is not publicly available.

The proportion of deaths of individuals in ICE detention attributable to suicide has approximately doubled since causes of death were last described for this population [7]. At least a third of individuals who died by hanging were noted to have been on suicide watch at some point during detention, and were therefore known to be at risk for death by suicide. As described in one of the illustrative case studies, one of the detained individuals, who died of a primary medical cause, had been recently discharged from a hospital against medical advice after reportedly refusing to cooperate with care for a diagnosed bowel obstruction while on suicide watch, raising questions about the detained individual's capacity to refuse care for a potentially life-threatening condition, and whether mental health issues may have been contributory to this death. Findings highlight that there is considerable room to improve access to and quality of psychiatric care as well as suicide prevention efforts for individuals in ICE detention.

COVID-19 has emerged as an important cause of mortality, responsible for nearly three quarters of deaths among individuals in ICE detention since April of 2020. The majority of COVID-19-related deaths occurred among detained individuals whose age [17] or medical conditions such as diabetes, chronic kidney disease, or lymphoma [18] placed them at high risk for severe illness or death from COVID-19. While transmission of COVID-19 and other respiratory illnesses such as influenza can be theoretically avoided with safety measures such as masks, hand hygiene, and social distancing, these measures prove difficult to implement in often-crowded detention environments. While COVID-19 vaccines were not available during the study period, the United States Food and Drug Administration recently issued an Emergency Use Authorization for the first COVID-19 vaccine with reported 95% effectiveness in preventing symptomatic laboratory-confirmed COVID-19 in persons without evidence of previous infection [19]. Immunizations for COVID-19 and other preventable illnesses such as influenza, which has also been responsible for many deaths of immigrants in detention, [20] should be prioritized for incarcerated populations—particularly for detained individuals whose age or co-morbid conditions place them at increased risk for mortality.

This study has several limitations. First, this study is limited to deaths occurring among individuals in ICE custody at the time of death during the study period, and does not include information on individuals (including children) who have died in the custody of United States Customs and Border Protection, [20] a separate federal agency specifically responsible for border management and control. These data also do not include individuals released by ICE just prior to death [10]. Thus, the calculated death rate likely underestimates deaths related to immigrant detention. Second, this was an exploratory study evaluating available information on ICE detention deaths over a three year period, and therefore, may have not been adequately powered to detect differences in characteristics between groups, though groups were compared using statistical tests as part of the descriptive exploration. Finally, death reports posted by ICE since the FY2018 mandate are less detailed than Detainee Death Reviews selectively released by ICE in response to Freedom of Information Act requests prior to 2018 [21]. The newer death reports are substantially shorter (2 to 5 pages compared to 12 to 167 pages) and present more limited information on circumstances leading up to death. However, this study represents an analysis of the best available data on deaths in ICE detention between FY2018 and FY2020.

## 5. Conclusions

There is considerable room for the improvement of infection control measures, access to and quality of psychiatric care and suicide prevention efforts for individuals in ICE detention. Greater detail in reporting on circumstances surrounding the deaths of individuals within ICE custody may be necessary to adequately address the observed increased death rate via evidence-based policies and improved external accountability.
