# Correction: Deaths in Immigration and Customs Enforcement (ICE) detention: FY2018–2020

**DOI:** 10.3934/publichealth.2021039

**Published:** 2021-07-07

**Authors:** Sophie Terp, Sameer Ahmed, Elizabeth Burner, Madeline Ross, Molly Grassini, Briah Fischer, Parveen Parmar

**Affiliations:** Keck School of Medicine, University of Southern California, Los Angeles, CA, USA

**A correction on**

Deaths in Immigration and Customs Enforcement (ICE) detention: FY2018–2020

By Sophie Terp, Sameer Ahmed, Elizabeth Burner, Madeline Ross, Molly Grassini, Briah Fischer, Parveen Parmar. Deaths in Immigration and Customs Enforcement (ICE) detention: FY2018–2020[J]. AIMS Public Health, 2021, 8(1): 81–89. doi: 10.3934/publichealth.2021006.

We would like to submit the following correction to our recently published paper due to the wrong of reference 8. The details as the following.

8. Congress.gov (2018) H. Rept. 115–239—Department OF Homeland Security Appropriations Bill, 2018. Available from: https://www.congress.gov/congressional-report/115th-congress/house-report/239.

