# Supplementary material for: Deaths in Immigration and Customs Enforcement (ICE) detention: FY2018–2020
Source: AIMS Public Health. 2021 Jan 11;8(1):81–9. doi: 10.3934/publichealth.2021006 (PMC7870381; doi:10.3934/publichealth.2021006)
Supplement: Supplementary file 1 [file publichealth-08-01-006-s001.pdf]

AIMS Public Health

http://www.aimspress.com/journal/aimsph

AIMS Public Health, 8(1): 81–89.
DOI: 10.3934/publichealth.2021006
Received: 12 November 2020
Accepted: 05 January 2021
Published: 11 January 2021

Research article

Deaths in Immigration and Customs Enforcement (ICE) detention: FY2018–2020

Sophie Terp*, Sameer Ahmed, Elizabeth Burner, Madeline Ross, Molly Grassini, Briah Fischer and Parveen Parmar

Keck School of Medicine, University of Southern California, Los Angeles, CA, USA

* **Correspondence:** Email: terp@usc.edu.

Supplementary

Supplementary Exhibit 1. Illustrative Case Study
In 2019, a 54-year-old citizen of Mexico was taken into ICE custody two decades after first entering the United States. At intake, he reported a history of diabetes, for which he was prescribed metformin, and he was diagnosed with restless leg syndrome, for which he was started on gabapentin. One month later, he was evaluated by an advanced practice provider for complaints of fever, chills, sore throat, and mild cough. The provider noted that the individual appeared ill, diagnosed influenza, and prescribed the antiviral medication oseltamivir, as well as acetaminophen as needed for fever or pain. An order was placed for housing in medical segregation with daily nursing checks. The next day at noon, nursing notes indicated "a normal physical exam, except a heart rate of 103 and pulse oximetry reading of 83%." Per documentation, a security officer informed the nurse that the individual had been lying in bed all day. The nurse encouraged the individual to be more active and educated him about hydration. Three hours later, the nurse reportedly advised the advance practice provider of pulse oximeter readings ranging from 79–83%. The nurse documented that the individual was ambulatory in his cell. From a review of available documentation, there was no indication that supplementary oxygen was ordered or administered at the time. The next morning, medical assistance was requested because the individual was having difficulty breathing, and by the time medical staff arrived, security staff had initiated cardiopulmonary resuscitation. A nurse documented that the individual was unconscious, unresponsive to any stimuli, had a weak, thready pulse, and that a reading from the pulse oximeter could not be obtained. Emergency medical services (EMS) personnel arrived twelve minutes later, noted that the individual was pulseless,

had no spontaneous respirations and appeared to have lividity. An electrocardiogram indicated the absence of a heartbeat. EMS pronounced death approximately 21 minutes after the medical emergency was identified. The medical examiner later documented the cause of death as complications of liver cirrhosis, diabetes mellitus, and hypertensive cardiovascular disease, and deemed the manner of death as natural.

Supplementary Exhibit 2. Illustrative case study.

A 55-year-old citizen of Russia was taken into ICE custody nearly two decades after entering the United States on a visitor visa, having overstayed the six-month visa without authorization. The detained individual's intake summary indicated a medical history of hypertension, an unspecified heart condition, and a seizure disorder. Records indicated a history of conversion disorder, though the individual reportedly denied a history of mental health conditions. A physician authorized the continuation of the individual's anti-hypertensive and anti-seizure medications. Three and a half months later, suicide watch protocols were initiated after security staff reported that the detained individual stated, "If I can't go home, then I just want to kill myself." One day later, a Licensed Mental Health Clinician discontinued suicide precautions and cleared the individual for a return to the general population. Over the next year, this individual was evaluated by mental health providers on a number of occasions, diagnosed with acute stress disorder, anxiety, insomnia and hallucinations, and prescribed chlorpromazine and hydroxyzine. Over the same yearlong period, this individual was evaluated for hypotension on three separate occasions over a three month period, and was advised to drink Gatorade each time without reported additional workup or intervention. On the fourth evaluation for hypotension three and a half months after the first, lab work was ordered, though results were not included in the death report. On the fifth evaluation for hypotension, approximately four months after the initial documented episode of hypotension, one of his anti-hypertensive medications was discontinued.

Four days prior to his death, approximately seventeen months after the individual entered ICE custody, he was found to be unresponsive and hypoxic (SaO_2 86%), and was transported to a local hospital where he was diagnosed with a small bowel obstruction and anemia. Per hospital staff, he reported that he would "rather die than to get better, and he wished to die." Two days later, he left the hospital against medical advice and was admitted to the detention facility's medical housing unit on suicide watch. No reference to assessment of capacity to refuse medical care was included in the report. The next morning, suicide watch protocols were discontinued after the individual denied suicidal ideation, though mental health observation with clothing and utensil restriction was implemented. Approximately four hours later, he was cleared for the general population without restrictions. Seven hours later, the individual was evaluated by a psychiatrist who diagnosed the individual with unspecified psychosis, and ordered that psychotropic medications be held due to his existing small bowel obstruction, with a request for psychiatric follow up to be scheduled in 30 days. At that time, the detained individual was reportedly experiencing shortness of breath and the psychiatrist requested that nurses consult with primary care regarding a possible x-ray. The next morning on evaluation in the medical housing unit, he was noted to be in visible distress with difficulty breathing, severe abdominal tenderness, lack of bowel sounds, pale skin, and a SaO_2 of 76%. He was transported to an emergency department by emergency medical services, where 12 minutes after arrival he reportedly vomited feces and went into cardiac arrest. He was pronounced dead after 48 minutes of unsuccessful resuscitation attempts.

AIMS Press

© 2021 the Author(s), licensee AIMS Press. This is an open access article distributed under the terms of the Creative Commons Attribution License (http://creativecommons.org/licenses/by/4.0)
